# Supplementary material for: Mutations in PCYT2 disrupt etherlipid biosynthesis and cause a complex hereditary spastic paraplegia
Source: Brain. 2019 Oct 22;142(11):3382–97. doi: 10.1093/brain/awz291 (PMC6821184; doi:10.1093/brain/awz291)
Supplement: awz291_Supplementary_Data [file awz291_supplementary_data.zip › awz291-Suppl_data/Supplementary_Data4.zip!/Supplementary information 4 - plasma lipidomics.zip!/Plasma lipidomics/Control vs Patient3a/Statistical plots and data/Heatmap sorted on p-value and FC.pdf]

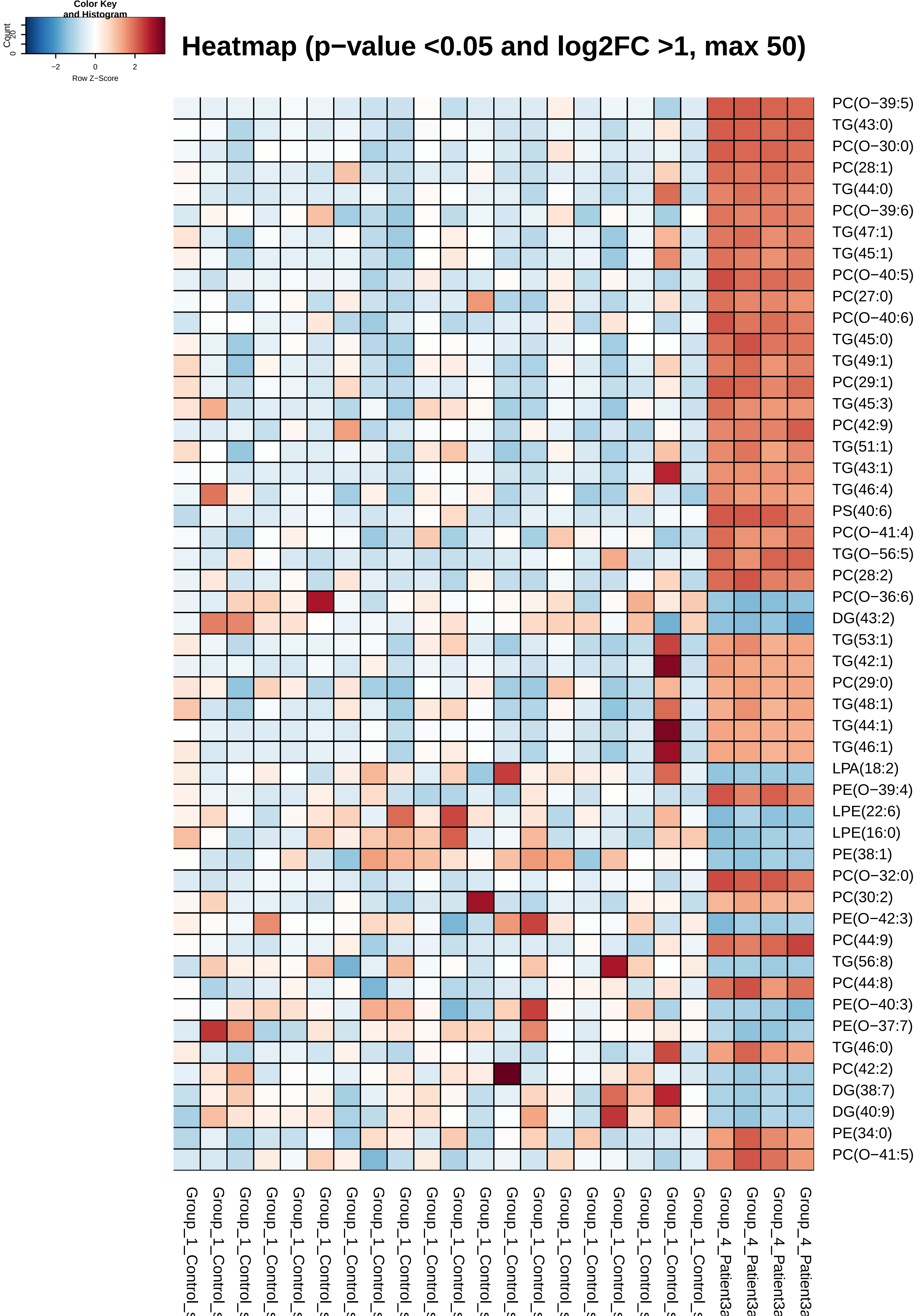
Color Key
and Histogram
Count
0
20
−2
0
2
Row Z−Score
Heatmap (p−value <0.05 and log2FC >1, max 50)
PC(O−39:5)
TG(43:0)
PC(O−30:0)
PC(28:1)
TG(44:0)
PC(O−39:6)
TG(47:1)
TG(45:1)
PC(O−40:5)
PC(27:0)
PC(O−40:6)
TG(45:0)
TG(49:1)
PC(29:1)
TG(45:3)
PC(42:9)
TG(51:1)
TG(43:1)
TG(46:4)
PS(40:6)
PC(O−41:4)
TG(O−56:5)
PC(28:2)
PC(O−36:6)
DG(43:2)
TG(53:1)
TG(42:1)
PC(29:0)
TG(48:1)
TG(44:1)
TG(46:1)
LPA(18:2)
PE(O−39:4)
LPE(22:6)
LPE(16:0)
PE(38:1)
PC(O−32:0)
PC(30:2)
PE(O−42:3)
PC(44:9)
TG(56:8)
PC(44:8)
PE(O−40:3)
PE(O−37:7)
TG(46:0)
PC(42:2)
DG(38:7)
DG(40:9)
PE(34:0)
PC(O−41:5)
Group_1_Control_s
Group_1_Control_s
Group_1_Control_s
Group_1_Control_s
Group_1_Control_s
Group_1_Control_s
Group_1_Control_s
Group_1_Control_s
Group_1_Control_s
Group_1_Control_s
Group_1_Control_s
Group_1_Control_s
Group_1_Control_s
Group_1_Control_s
Group_1_Control_s
Group_1_Control_s
Group_1_Control_s
Group_1_Control_s
Group_1_Control_s
Group_1_Control_s
Group_4_Patient3a
Group_4_Patient3a
Group_4_Patient3a
Group_4_Patient3a
